# Complete Genome Sequence of *Alstroemeria* *Necrotic Streak Virus* Isolated from Limonium sinuatum in Ecuador

**DOI:** 10.1128/mra.00622-22

**Published:** 2022-10-18

**Authors:** Marco Taipe, Francisco Flores

**Affiliations:** a Centro de Estudios de Posgrado de la Universidad de las Fuerzas Armadas, Sangolquí, Ecuador; b Departamento de Ciencias de la Vida y la Agricultura, Universidad de las Fuerzas Armadas, Sangolquí, Ecuador; c Centro de Investigación de Alimentos, Facultad de Ciencias de la Ingeniería e Industrias, Universidad UTE, Quito, Ecuador; DOE Joint Genome Institute

## Abstract

The complete genome sequence of *Alstroemeria* *necrotic streak virus* (ANSV) isolated from the statice cut flower (Limonium sinuatum) is presented. The genome has the three characteristic segments of the genus *Orthotospovirus*, family *Tospoviridae*, the S segment (3,135 bp), the M segment (4,869 bp), and the L segment (8,755 bp).

## ANNOUNCEMENT

An important agricultural activity in the Ecuadorian highlands is floriculture. Limonium sinuatum (L.) Mill is a cut flower species considered a complement for fresh bouquets (1); although its cultivation is mostly done by large floricultural companies, the crop is also important for the economy of small and medium-size farmers. In January 2020, statice leaf samples with symptoms of virus infection, including leaf malformation, dwarfism, and streaking, were collected from a flower plantation located in El Quinche, a parish close to Quito, Ecuador.

Total RNA was extracted from samples of symptomatic leaves using the SV total RNA isolation system (Promega). Sequencing was performed by Macrogen Inc. (Seoul, South Korea) with a NovaSeq 6000 sequencer (Illumina Inc., USA), and libraries were prepared with the TruSeq stranded total RNA with Ribo-Zero plant kit, generating two files with 26,130,229 paired-end reads. The reads were trimmed and filtered using Trimmomatic v0.39 (2) (http://www.usadellab.org/cms/index.php?page=trimmomatic), duplicated sequences were removed with the BBMap v38.82 tool (3), generating 7,036,551 reads between 40 and 91 bp in length, and the final assembly was performed with SPAdes v3.13.1 (4) (https://cab.spbu.ru/software/spades). The contigs were compared with plant virus reference sequences from NCBI GenBank using BLASTn (5) (https://blast.ncbi.nlm.nih.gov/Blast.cgi). Four contigs, between 1,311 and 9,047 bp in size, matched reference sequences for the *Alstroemeria* *necrotic streak virus* (ANSV) segments (GenBank accession number NC_055298 for segment L, GenBank accession number NC_055297 for segment M, and GenBank accession number NC_055299 for segment S). Two contigs were used to assemble the S segment by mapping them to the reference sequence using Geneious v6.1.8, while the other two contigs represented the complete L and M segments.

The final assembled sequences had the following features: a length of 8,755 bp, with a GC content of 34.2%, depth of coverage of 890×, and 98.9% identity with the reference sequence, for the L segment; a length of 4,869 bp, with a GC content of 35.6%, depth of coverage of 2,870×, and 98.7% identity with the reference sequence, for the M segment; and a length of 3,135 bp, with a GC content of 35.1%, depth of coverage of 3,500×, and 99.3% identity with the reference sequence, for the S segment. The ANSV reference genome has reported lengths of 8,756 bp, 4,839 bp, and 3,113 bp for the L, M, and S segments, respectively, which suggests that the complete genome was obtained.

Open reading frame (ORF) prediction was performed with the Geneious v6.1.8 program. The L segment has one ORF, encoding the L protein (8,625 bp). The M segment has two ORFs; ORF1 encodes the nonstructural protein (912 bp), and ORF2 encodes the precursor glycoprotein (3,381 bp). The S segment has two ORFs; ORF1 encodes a nonstructural protein (1,404 bp), and ORF2 encodes the nucleocapsid protein (777 bp).

**FIG 1:**
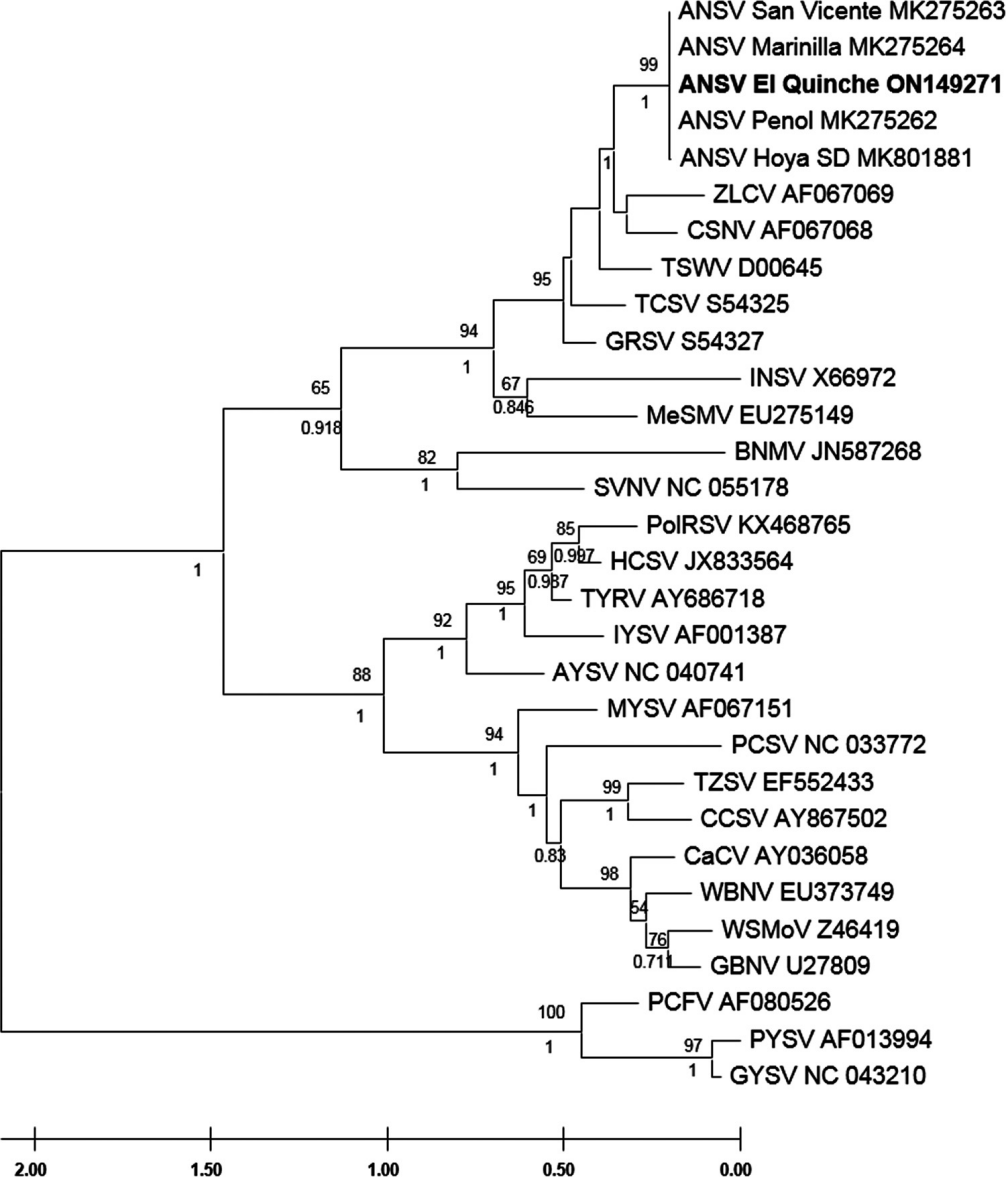
Maximum likelihood phylogenetic tree for the nucleocapsid sequence of orthotospoviruses, including the ANSV El Quinche strain. Bootstrap support values are shown above the nodes, and posterior probability values are shown below the nodes. Virus acronyms are: *Alstroemeria necrotic streak virus (ANSV), strains San Vicente, Marinilla, El Quinche, Penol, and Hoya SD; Zucchini lethal chlorosis virus (ZLCV); Chrysanthemum stem necrosis virus (CSNV); Tomato spotted wilt virus (TSWV); Tomato chlorotic spot virus (TCSV); Groundnut ringspot virus (GRSV); Impatiens necrotic spot virus (INSV); Melon severe mosaic virus (MeSMV); Bean necrotic mosaic virus (BNMV); Soybean vein necrosis virus (SVNV); Polygonum ringspot virus (PolRSV); Hippeastrum chlorotic spot virus (HCSV); Tomato yellow ring virus (TYRV) ; Iris yellow spot virus (IYSV); Alstroemeria yellow spot virus (AYSV); Melon yellow spot virus (MYSV); Pepper chlorotic spot virus (PCSV); Tomato zonate spot virus (TZSV); Calla lily chlorotic spot virus (CCSV); Capsicum chlorosis virus (CaCV); Watermelon bud necrosis virus (WBNV); Watermelon silver mottle virus (WSMoV); Groundnut bud necrosis virus (GBNV); Peanut chlorotic fan-spot virus (PCFV); Peanut yellow spot virus (PYSV); Groundnut yellow spot virus (GYSV)*. The GenBank accession numbers are at the end of each virus acronym.

Based on the amino acid sequence of the nucleocapsid, phylogenetic inference was performed for the El Quinche ANSV strain and 29 other *Orthotospovirus* sequences available in GenBank. Maximum likelihood analysis was performed using MEGA-X v10.2.6 (6) (www.megasoftware.net) with 1,000 bootstrap pseudoreplicates, and Bayesian probability was inferred with MrBayes v3.2.7 (7) (https://nbisweden.github.io/MrBayes/download.html). The El Quinche ANSV was placed in the same group as previously reported ANSV strains from Colombia (8), with bootstrap support values of 99 and posterior probability of 1 (Fig. 1). All tools were run with default parameters unless otherwise specified.

### Data availability.

Sequences of the ANSV virus segments found in El Quinche, Ecuador, are available in GenBank with accession numbers ON149271 (ANSV segment S), ON149272 (ANSV segment M), and ON149273 (ANSV segment L). SRA data are available under BioProject accession number PRJNA880414.
